# Revolutionizing Back Pain Management: Is Epidural Platelet-Rich Plasma the Superior Choice Over Steroids?

**DOI:** 10.7759/cureus.55423

**Published:** 2024-03-03

**Authors:** Adarsh Jayasoorya, Nitin Samal, Gajanan Pisulkar, Ankur Salwan, Kevin Kawde

**Affiliations:** 1 Department of Orthopaedics, Jawaharlal Nehru Medical College, Datta Meghe Institute of Higher Education and Research, Wardha, IND

**Keywords:** epidural steroid, epidural prp, low back pain, straight leg raising test, visual analogue scale (vas) score, oswestry disability questionnaire score, discogenic pain

## Abstract

Background and objective

Low back discomfort is one of the main factors that restrict physical activity, and it is becoming more and more common. Surgery is the best option when all other conservative treatment methods have failed, but it is not a panacea. While local anesthetic-free and combined epidural steroid injections have been used for many years, their usefulness is limited to shorter periods. In the field of orthopedics, platelet-rich plasma (PRP) has gained widespread recognition as an adjuvant component. PRP has been applied to improve tissue repair, both soft and hard. This comparative study aimed to evaluate the potential of PRP as a therapy for low back pain (LBP).

Methods

We included 64 adult individuals with complaints of LBP. They were classified into two groups: group A underwent a single injection in the afflicted lumbar intervertebral disc (IVD) level with 1.5 ml of methylprednisolone, 1.5 ml 2% lidocaine, and 0.5 ml of saline under rigorous aseptic precautions; in contrast, group B was administered a single injection of 3 milliliters of autologous PRP. Patients' scores on the visual analog scale (VAS), the Modified Oswestry Disability Questionnaire (MODQ), and the Straight Leg Raising Test (SLRT) were assessed before and during therapy.

Results

The data gathered were subjected to statistical analysis. Statistically significant differences were found in the VAS scores between group A (methylprednisolone group) and group B (PRP group) post-one hour (6.0 ±0.74 vs. 6.92 ±0.57) and after three months (5.2 ±0.65 vs. 3.26 ±0.79).

Conclusions

Our study revealed gradual progressive improvement in the symptoms of patients in the PRP group as indicated by scores on SLRT, VAS, and MODQ. The results were comparable to those who received methylprednisolone injections. There was a statistically significant difference in VAS scores between the two groups, with the PRP group reporting a higher degree of pain reduction, showing that PRP is an effective alternative to epidural steroid infiltration in managing chronic LBP.

## Introduction

One of the main causes of physical handicap that affects both younger and older adults is low back pain (LBP), which can have significant adverse effects on one's health and socioeconomic situation. Age-related degeneration of the intervertebral disc (IVD), which impacts the nerve system around the disc, is the main cause of LBP. Discogenic pain is the pain that occurs from excitation of the nociceptors in the annulus fibrosus [1]. This kind of discomfort is becoming epidemic in scope and is one of the primary causes of limitations in physical activity among many individuals [2].

There are two main approaches to treating lumbar IVD degeneration: surgery and conservative measures. The major goal of conservative treatment for LBP is to lessen pain perception. This is achieved through rest, physical therapy, and anti-inflammatory medications. By removing the motion between spinal segments, intervertebral fusion surgery aims to reduce discogenic pain [3]. When conservative treatment approaches are exhausted, surgery is the next best method, but it is not a cure-all. Although epidural steroid injections with local anesthetics and without local anesthetics have been utilized for several years, their effectiveness is restricted to shorter durations [4]. Platelet-rich plasma (PRP) has become well-known as an adjuvant component in the field of orthopedics [5]. Both soft-tissue and hard-tissue healing are enhanced by PRP [6,7,8,9]. Innovative therapeutic approaches are emerging, focusing on the biological events in the degenerative process to avoid the need for invasive spinal surgeries [10]. The current comparative study was designed to ascertain the potential use of PRP in treating LBP.

## Materials and methods

Study design, setting, ethical consideration, and participants

A total of 64 adults with LBP who reported to the orthopedic department at Acharya Vinoba Bhave Rural Hospital (AVBRH), Sawangi, Wardha were included in the current prospective randomized study. Ethical clearance was obtained from the institutional ethical committee (ethics approval ref. no. DMIMS (DU)/IEC/2022/47 dated 18/07/2022). All patients provided written informed permission following an explanation of the procedure.

Study criteria (inclusion and exclusion)

All skeletally mature individuals with lumbar IVD prolapse diagnosed with LBP lasting at least four weeks and a positive Straight Leg Raising Test (SLRT) were included if they met the inclusion criteria [10]. The exclusion criteria were as follows: bleeding disorders, infections, serious spine injuries, tumors, malformation, and congenital spinal defects.

Sample size

Patients who satisfied the requirements for participation were split into two groups: group A and group B. The patients were divided into 32 groups using a block randomization procedure using computer-generated random numbers. A system of sequentially numbered opaque sealed envelopes was used to implement the sequence of allocation. The allocation sequence was created by the primary investigator, who oversaw participant recruitment and intervention assignment as well.

Preparation of PRP 

A standard noncooled REMI R-8C centrifuge type was used to produce autologous PRP by using the standard double spin procedure. The process was performed in a sterile environment at an ambient temperature of 24°C; 20 ml of blood was taken from the patient's median cubital vein and split among four sterile vials with sodium citrate. These vials were centrifuged at 2000 rotations per minute for 15 minutes. Whereas plasma gathered in the upper part of the test tube, RBCs settled in the lower half. After extraction, the plasma was collected in a different test tube. It underwent further centrifugation for 10 minutes at 1200 rotations per minute. The platelet-poor plasma (PPP) and PRP were separated. While PPP was gathered in the top buffy coat, PRP was retrieved in the lowest 2-4 ml layer of the plasma. This autologous PRP was the standard regenerative biological product.

Data collection and patient management

The laboratory performed X-rays, coagulation profile, CBC, ESR, blood sugar level, and lateral and anteroposterior views of the lumbosacral spine before the start of treatment. Six hours before surgery, participants' scores were kept at 0 on the oral portion of the Modified Oswestry Disability Questionnaire (MODQ), and their score on the VAS was maintained [11,12,13]. Before the surgery, SLRT results were recorded [14]. Group A underwent a single injection at the afflicted lumbar IVD level with 1.5 ml of methylprednisolone, 1.5 ml of 2% lidocaine, and 0.5 ml of saline under rigorous aseptic precautions. In contrast, group B was administered a single injection of 3 milliliters of autologous PRP, which was prepared under sterile and aseptic settings, adhering to rigorous guidelines; 20 milliliters of blood was withdrawn from the patient's median cubital vein, which was centrifuged to produce 3 milliliters of PRP. Using an 18G needle and an interlaminar technique, 3 ml of autologous PRP was injected into the epidural space under extreme aseptic conditions.

Outcome measures

Patients were asked to stop using all analgesics, including non-steroidal anti-inflammatory drugs (NSAIDs), two weeks before the procedure. The patient's hemodynamic parameters, including temperature, pulse rate, oxygen saturation, and blood pressure, were monitored every 10 minutes for 30 minutes after the surgery and were also observed for any issues. They were evaluated and the outcomes of the SLRT, VAS, and MODQ were noted one hour after the surgery. Patients were told not to move heavy things, bend over a lot, or walk for long periods after discharge. They were also asked to follow up at three weeks and three months. Continuous records of the MODQ, VAS, and SLRT scores were kept.

Statistical analysis

Data obtained were statistically analyzed by using SPSS Statistics version 21 (IBM Corp., Armonk, NY), and the Mann-Whitney U test was applied.

## Results

In terms of VAS ratings, MODQ scores, and SLRT, study subjects who received autologous PRP injections via lumbar epidural demonstrated a greater improvement in the symptoms. The patients' symptoms continued to gradually improve for three months until they were assessed. Additionally, there was a significant variation in the VAS scores between both groups, with the PRP group experiencing a greater reduction in pain. As shown in Table 1, the results revealed that the mean VAS score at baseline was 7.06 ±0.98 in group A and 7.06 ±0.98 in group B with a non-significant difference. Post-one hour, it was 6.0 ±0.74 in group A and 6.9 2±0.57 in group B, which was statically significant (p<0.05). On follow-up after three weeks, it was 5.85 ±0.88 in group A and 5.43 ±1.6 in group B, which was non-significant. After three months, it was 5.2 ±0.65 in group A and 3.26 ±0.79 in group B, which was statistically significant (p<0.05).

**Table 1:** Visual analog scale (VAS) scores PRP: platelet-rich plasma; SD: standard deviation

	Group A (methylprednisolone group), n=32, mean ±SD	Group B (PRP group), n=32, mean ±SD	t value	P-value
Baseline	7.06 ±0.98	7.21 ±1.31	-0.15	Not significant
Post one hour	6.0 ±0.74	6.92 ±0.57	0.92	<0.05
After three weeks	5.85 ±0.88	5.43 ±1.6	0.42	Not significant
After three months	5.2 ±0.65	3.26 ±0.79	1.94	<0.05

Table 2 shows the MODQ scores. In group A, in the 0% to 20% score criteria (minimal disability), post-procedure after one hour, one patient was found to fulfill these criteria, six subjects after three weeks, and four subjects after three months. In group B, five subjects were found to fulfill these criteria after three weeks and five subjects after three months. In the 21% to 40% scoring criteria (moderate disability), in group A, three subjects were in this category pre-procedurally, 10 subjects post-procedure after one hour, seven subjects after three weeks, and 10 subjects after three months; in group B, five subjects fell in this criteria pre-procedurally, eight subjects after one hour, 16 subjects after three weeks and 18 subjects after three months. In the 41% to 60% scoring criteria, (severe disability), in group A, 27 subjects were found to fulfill this pre-procedurally, 20 subjects post-procedure after one hour, 19 subjects after three weeks, and 18 subjects after three months; in group B, 23 subjects fell in this criteria pre-procedurally, 21 subjects after one hour, 11 subjects after three weeks, and nine subjects after three months. In the 61% to 80% scoring criteria, (crippled), in group A, two subjects were in this category pre-procedurally and one subject post-procedure after one hour; in group B, four subjects fell in this criteria pre-procedurally and three subjects after one hour. This reveals a gradual improvement in the MODQ score.

**Table 2:** Modified Oswestry Disability Questionnaire (MODQ) scores PRP: platelet-rich plasma

	Group A (corticosteroid group), n=32				Group B (PRP group), n=32			
	Baseline (before procedure)	Post-procedure after one hour	After three weeks	After three months	Baseline (before procedure)	Post-procedure after one hour	After three weeks	After three months
0% to 20% (minimal disability)	0	1	6	4	0	0	5	5
21% to 40% (moderate disability)	3	10	7	10	5	8	16	18
41% to 60% (severe disability)	27	20	19	18	23	21	11	9
61% to 80% (crippled)	2	1	0	0	4	3	0	0
81% to 100% (bed-bound)	0	-	-	-	0	-	-	-

Table 3 shows the SLRT scores; in group A, 17 subjects showed up to 35-degree SLRT scores at baseline, 17 subjects post-procedure after one hour, and one subject after three weeks; in group B, 16 subjects fulfilled this at baseline and 19 subjects after one hour. As for 35-70 degree SLRT scores, in group A, 15 subjects showed this at baseline, 15 subjects post-procedure after one hour, 21 subjects after three weeks, and 16 subjects after three months; in group B, 16 subjects fell in this category at baseline, 13 subjects after one hour, 25 subjects after three weeks, and 15 subjects after three months. Regarding, the beyond-70-degree SLRT score category, in group A, 10 subjects were in it after three weeks and 16 subjects after three months; in group B, seven subjects showed this after three weeks and 17 subjects after three months. This increase in scoring reveals a gradual improvement in SLRT criterion and better treatment outcomes.

**Table 3:** Straight Leg Raising Test (SLRT) scores Pain up to 35 degrees is diagnostic of PIVD; 35-70 degrees is suggestive of disc prolapsed; pain beyond 70 degrees is equivocal PRP: platelet-rich plasma; PIVD: prolapsed intervertebral disc

	Group A (corticosteroid group), n=32				Group B (PRP group), n=32			
	Baseline (before procedure)	Post-procedure after one hour	After three weeks	After three months	Baseline (before procedure)	Post-procedure after one hour	After three weeks	After three months
Upto 35 degrees	17	17	1	0	16	19	0	0
35 to 70 degrees	15	15	21	16	16	13	25	15
Beyond 70 degrees	-	-	10	16	0	0	7	17

## Discussion

LBP remains one of the most prevalent and difficult health problems that affects the daily lives of individuals. It causes significant financial burdens, such as missed workdays and income; medical, surgical, and rehabilitation expenses; as well as the costs of incapacitating pain and restricted daily activities [15,16]. In our study, according to VAS ratings, MODQ scores, and SLRT, subjects who received autologous PRP injections via lumbar epidural injection demonstrated a progressive improvement in their symptoms. These findings were compared to the improvement shown with methylprednisolone injections. The patients' symptoms continued to gradually improve for three months until they were assessed. Furthermore, a noteworthy distinction was observed in the VAS ratings between the two groups, with the PRP group experiencing a greater reduction in pain.

In line with our research, Verma et al. found that patients' symptoms gradually improved over three months, no problems developed, and they were able to go about their everyday lives without the need for painkillers [1]. In a related trial, Wongjarupong et al. reported that patients receiving PRP injections had comparable outcomes on other fronts and a significant decrease in leg VAS scores at six, 12, and 24 weeks as well as in Oswestry Disability Index (ODI) at 24 weeks [5]. Outcomes from non-commercial epidural double-spin PRP injections were better than those from triamcinolone. For the treatment of single-level lumbar herniated nucleus pulposus (HNP), surgery is advised due to its effectiveness and safety. Similar to the current study, Bhatia et al. administered 5 ml of autologous PRP via epidural injection into the affected nerve root area under fluoroscopic guidance [2]. Up until the patients were under observation after three months, a progressive improvement was observed in their symptoms. At three months, the majority of patients had improved, with a VAS score of 4 or below. At three months, the majority of patients' SLRT improved to >70 and their MODQ score was less than 30%.

Three cases of LBP in elderly individuals treated with lumbosacral PRP injection were presented by Tolbert et al. The number of injections varied based on the patient's needs and was administered a few weeks apart. The patients' pain scores decreased from 8.0 to 1.5, 5.0 to 3.5, and 7.0 to 2.0, respectively [16]. In one case, the outcome was pain-free ambulation; in the second, the result was better sitting; and in the third, it was independent community walking. Moreover, angiogenesis, local immunity, growth factor levels, and stem cell count are among the fundamental healing mechanisms that are suppressed with age; as a result, nonhealing wounds are increasingly common as people age. PRP therapy may help older patients with nonhealing or delayed wounds heal more quickly after surgery [17]. PRP is an autologous variation of fibrin glue that has been reported and utilized in several applications with apparent clinical success [18]. It is produced using techniques that concentrate autologous platelets. There are 50-80 alpha granules with around 30 bioactive proteins in each platelet. Hemostasis and the healing of both soft and hard tissues depend on these proteins. A platelet count between 150,000 and 300,000/microL is regarded as typical. It is an easy-to-access source of growth factors that aid in bone and soft tissue repair. Platelet concentration or natural blood clotting may be easily enhanced using PRP. Therefore, to provide growth elements to the site at a greater concentration, PRP derived from autologous blood is utilized [18].

Pro-inflammatory cytokines and inflammation-related chemicals, such as neurotrophic factors, are thought to be behind the pathophysiology of discogenic LBP during the disc degeneration process. It has been demonstrated that neurotrophic factors are important in the transmission of both pathological and normal pain. Among them, the neurotrophin family is known to contribute to pain transmission and inflammatory responses by upregulating the expression of peptides associated with pain in response to inflammation in the surrounding tissue [19]. Growth factors, chemokines, and cytokines included in PRP preparations trigger downstream signaling pathways that ultimately result in the creation of proteins required for both the stimulation of structural and functional healing as well as the reduction of pain perception.

The healing process is accelerated by the higher platelet count because it causes an increase in growth factors to be released. This phenomenon is explained by its promotion of angiogenesis in the tissues and the mitogenesis of healing-capable cells. PRP preparations have been linked to better sleep and a reduction in narcotic use in addition to their role in structural and functional repair [20]. Using anterograde axon tracing, a quantitative assessment of the axon development from the brain cortex into the spinal cord was conducted by Takeuchi et al. They discovered that TGF-β1 in PRP has a detrimental effect on axon growth and that PRP stimulates axon growth in spinal cord tissues through pathways connected to VEGF and IGF-1 [21]. One of the shortcomings of the current treatment modality is that certain patients were not informed that a blood sample would be obtained, and hence they were not ready for treatment. Patients should therefore be informed about the benefits and reassured that the operation is being performed in an aseptic environment before the surgery.

Limitations 

This study has a few limitations, such as the gradual amelioration of pain symptoms observed in the PRP group when contrasted with the steroid group. Individuals experiencing acute pain might not attain immediate relief and may require supplementation with other oral or injectable pain relievers. Additionally, some patients may express reluctance to contribute their blood for the procedure.

## Conclusions

Based on our findings, patients who received autologous PRP injection through the lumbar epidural route experienced a gradual progressive improvement in their symptoms as indicated by improved scores on SLRT, VAS, and MODQ. The results were comparable to those of methylprednisolone injection. However, the VAS scores of the two groups differed in a statistically significant manner, with the PRP group reporting a higher degree of pain reduction in the long term (three months). The patients' symptoms showed a steady improvement for three months. Thus, it is evident that PRP is a feasible, secure, and efficacious therapeutic approach in the treatment of LBP.

## References

[1] Verma R, Mathur M, Prakash V, Kaushik SK (2020). Role of platelet-rich plasma (PRP) in chronic prolapsed intervertebral disc. Int J Orthop Sci.

[2] Bhatia R, Chopra G (2016). Efficacy of platelet-rich plasma via the lumbar epidural route in chronic prolapsed intervertebral disc patients-a pilot study. J Clin Diagn Res.

[3] Zhang J, Liu D, Gong Q, Chen J, Wan L (2022). Intradiscal autologous platelet-rich plasma injection for discogenic low back pain: a clinical trial. Biomed Res Int.

[4] Singh GK, Talawar P, Kumar A, Sharma RS, Purohit G, Bhandari B (2023). Effect of autologous platelet-rich plasma (PRP) on low back pain in patients with prolapsed intervertebral disc: a randomised controlled trial. Indian J Anaesth.

[5] Wongjarupong A, Pairuchvej S, Laohapornsvan P, Kotheeranurak V, Jitpakdee K, Yeekian C, Chanplakorn P (2023). "Platelet-Rich Plasma" epidural injection an emerging strategy in lumbar disc herniation: a randomized controlled trial trial. BMC Musculoskelet Disord.

[6] Sybil D, Sawai M, Faisal M, Singh S, Jain V (2020). Platelet-rich fibrin for hard- and soft-tissue healing in mandibular third molar extraction socket. Ann Maxillofac Surg.

[7] O'Connell SM, Impeduglia T, Hessler K, Wang XJ, Carroll RJ, Dardik H (2008). Autologous platelet-rich fibrin matrix as cell therapy in the healing of chronic lower-extremity ulcers. Wound Repair Regen.

[8] Doss AX (2012). Trigeminal neuralgia treatment: a case report on short-term follow up after ultrasound-guided autologous platelet-rich plasma injections. WebmedCentral.

[9] Anjayani S, Wirohadidjojo YW, Adam AM, Suwandi D, Seweng A, Amiruddin MD (2014). Sensory improvement of leprosy peripheral neuropathy in patients treated with perineural injection of platelet-rich plasma. Int J Dermatol.

[10] Navani A, Ambach M, Calodney A, Rosenthal R, Li G, Mahoney CB, Everts PA (2024). The safety and effectiveness of orthobiologic injections for discogenic chronic low back pain: a multicenter prospective, crossover, randomized controlled trial with 12 months follow-up. Pain Physician.

[11] Haefeli M, Elfering A (2006). Pain assessment. Eur Spine J.

[12] Fairbank JC, Pynsent PB (2000). The Oswestry Disability Index. Spine (Phila Pa 1976).

[13] Davidson M, Keating JL (2002). A comparison of five low back disability questionnaires: reliability and responsiveness. Phys Ther.

[14] Willhuber GO, Piuzzi NS (2023). Straight Leg Raise Test. StatPearls.

[15] Heneweer H, Staes F, Aufdemkampe G, van Rijn M, Vanhees L (2011). Physical activity and low back pain: a systematic review of recent literature. Eur Spine J.

[16] Takeuchi M, Kamei N, Shinomiya R (2012). Human platelet-rich plasma promotes axon growth in brain-spinal cord coculture. Neuroreport.

[17] Tolbert G, Roy D, Walker V (2013). Ultrasound guided dextrose prolotherapy and platelet rich plasma therapy in chronic low back pain: three case reports. Int J Phys Med Rehabil.

[18] Davis VL, Abukabda AB, Radio NM, Witt-Enderby PA, Clafshenkel WP, Cairone JV, Rutkowski JL (2014). Platelet-rich preparations to improve healing. Part I: workable options for every size practice. J Oral Implantol.

[19] Saluja H, Dehane V, Mahindra U (2011). Platelet-Rich fibrin: A second generation platelet concentrate and a new friend of oral and maxillofacial surgeons. Ann Maxillofac Surg.

[20] Akeda K, Yamada J, Linn ET, Sudo A, Masuda K (2019). Platelet-rich plasma in the management of chronic low back pain: a critical review. J Pain Res.

[21] Ramaswamy Reddy SH, Reddy R, Babu NC, Ashok GN (2018). Stem-cell therapy and platelet-rich plasma in regenerative medicines: A review on pros and cons of the technologies. J Oral Maxillofac Pathol.

